# Prenatal iodine intake and infant temperament in a multiethnic US cohort

**DOI:** 10.1017/S1368980024001575

**Published:** 2024-11-06

**Authors:** Aderonke A Akinkugbe, Julia Duffy, Srimathi Kannan, Terryl J Hartman, Julio Landero, Michelle Bosquet Enlow, Robert O Wright, Xueying Zhang, Rosalind J Wright

**Affiliations:** 1 Department of Environmental Medicine and Climate Science, Icahn School of Medicine at Mount Sinai, 1 Gustave L. Levy Pl, New York, NY 10029, USA; 2 Institute for Climate Change, Environmental Health and Exposomics, Icahn School of Medicine at Mount Sinai, New York, NY, USA; 3 Tufts University School of Medicine, Boston, MA, USA; 4 Division of Metabolism, Endocrinology, and Diabetes, Department of Internal Medicine, University of Michigan, Ann Arbor, MI, USA; 5 Department of Epidemiology, Rollins School of Public Health, Emory University, Atlanta, GA, USA; 6 Department of Psychiatry and Behavioral Sciences, Boston Children’s Hospital and Department of Psychiatry Harvard Medical School, Boston, MA, USA; 7 Department of Public Health, Icahn School of Medicine at Mount Sinai, New York, NY, USA

**Keywords:** Iodine intake, Pregnancy, Infant temperament, Sex-specific, Pregnancy cohort

## Abstract

**Objective:**

Maternal iodine plays a central role in fetal neurodevelopment. It is recommended that pregnant women consume sufficient levels of iodine to accommodate increased need for mother and fetus. We examined associations among prenatal dietary and supplemental iodine intake and infant temperament.

**Design:**

The PRogramming of Intergenerational Stress Mechanisms (PRISM) study is an ongoing longitudinal pregnancy cohort. Data from 2011 to 2020 were used for this study. Women completed the Block98 FFQ ascertaining prenatal dietary and supplemental iodine intake and the Infant Behavior Questionnaire-Revised at infant age 6 months to ascertain infant temperament (Surgency/Extraversion, Negative Affectivity and Orienting/Regulation).

**Setting:**

USA.

**Participants:**

Mother–child dyads (*n* 892).

**Results:**

Women were primarily Black/Hispanic Black (44 %) and non-Black Hispanic (35 %) with 46 % reporting household income < $25 000/year. Nearly half had an estimated average requirement (EAR) < 160 µg/d (49 % based on dietary intake; 43 % based on diet and supplements). Girls born to women with an EAR ≥ 160 µg/d compared to girls born to women below this level had higher unadjusted extraversion scores for dietary plus supplemental intake (*β* = 0·23 (0·13, 0·33)); decreased to *β* = 0·05 (–0·08, 0·19) after adjusting for covariates. Boys born to women with an EAR ≥ 160 µg/d (based on diet and supplements) as compared to boys born to women below this level had statistically non-significant higher unadjusted negative affective score (*β* = 0·06 (–0·08, 0·20)) that became significantly lower upon covariate adjustment (*β* = –1·66 (–1·97, –1·35)).

**Conclusions:**

A significant proportion of these women reported suboptimal prenatal iodine intake. Suboptimal prenatal iodine intake may have implications for child neurodevelopment evident as early as infancy.

Iodine is biochemically involved in the production of thyroid hormones, such as thyroxine (T4) and triiodothyronine (T3), which play important roles in neurological development, metabolism, growth and reproduction^(1,2)^. Optimal thyroid hormone production may be particularly relevant during pregnancy, as maternal T4 activates genes that regulate fetal brain development^(3)^. Suboptimal iodine levels during gestation also contribute to oxidative stress, with consequent disturbances in placental redox balance, which is central to optimal fetal development, including the brain^(4)^. It is thus recommended that pregnant women consume sufficient levels of iodine to accommodate the increased need for both mother and fetus^(2,5)^.

The impact of severe iodine deficiency in pregnancy on fetal brain development is well established^(1)^, emerging epidemiological studies, largely outside of the USA, document consequences of mild-to-moderate deficiencies. Indeed, inadequate iodine intake is a growing public health issue in the USA and other industrialised countries, in part due to changing dietary patterns and food manufacturing practices^(6)^. For example, changes to commercial processing techniques for milk and bread^(7)^ have led to a decrease in iodine intake^(3,8)^. Moreover, surveillance data from the US documents a worrisome trend among pregnant women. Data from the US National Health and Nutrition Examination Survey (NHANES) 2005–2010 reported a median urinary iodine concentration (UIC) of 129 μg/l (95 % CI 101, 173) in pregnant women^(9)^, significantly lower than in 2001–2006 (153 μg/l, 95 % CI 105, 196)^(10,11)^. Non-Hispanic Black pregnant women were particularly likely to have iodine levels below 150 µg/l, the median UIC for pregnant women recommended by the WHO^(12)^.

A recent scoping review summarised epidemiological studies from Europe, India, Japan and Scandinavian countries that have examined associations between gestational iodine intake and measured levels in relation to child neurodevelopmental outcomes and reported mixed results^(13)^. The Norwegian Mother and Child Cohort Study using data on 33 047 mother–child pairs found that children born to women with inadequate iodine intake levels during pregnancy were more likely to score lower on tests of verbal IQ, reading accuracy, reading comprehension and spelling^(3)^. Specifically, iodine intake below the dietary reference intakes estimated average requirement (EAR) of 160 µg/d of iodine for pregnant women was associated with language delay, behaviour problems and reduced fine motor skills at the age of 3 years^(3)^. In this same population, investigators also linked low dietary iodine intake during pregnancy with higher attention-deficit hyperactivity disorder (ADHD) symptom scores among 8-year-olds^(8)^. Studies examining associations between prenatal iodine status and child neurodevelopmental outcomes in US samples are sparse, particularly among racially/ethnically diverse women and children. Moreover, much of this literature considers outcomes in older children. Research on early outcomes in infancy and preschool-aged children is needed to identify risk as early in development as possible.

Temperament, measurable beginning in infancy, encompasses individual variations in behavioural tendencies of emotional responses and reactions to internal, social and environmental stimuli and comprises various domains that reflect relatively stable traits over an individual’s lifespan^(14)^. Specifically, temperament has been characterised as involving two reactive dimensions (i.e. negative affectivity and extraversion/surgency) and one regulatory dimension (i.e. orienting/regulation in infancy, effortful control in childhood). Infant temperament is associated with later personality and social development and risk for emotional and behavioural problems^(15)^. For example, orienting/regulation has been positively associated with more optimal executive control and executive function in preschool and later childhood periods^(16)^. Increased negative affectivity has been linked to persistent difficulties, including internalising problems^(17)^ in adolescence and later life^(18)^, highlighting the need to explicate potentially modifiable environmental risk factors that contribute to early-life behavioural domains so that interventions can be applied early to promote optimal development.

Although the association between infant temperament difficulties and later psychopathology is robust, the magnitude of effects can be variable^(19,20)^. This may be due, in part, to how children respond to similar environmental challenges in divergent ways, with individual characteristics of the child, including sex, influencing pathways to adaptive or maladaptive outcomes. A few early developmental studies have associated prenatal dietary patterns or specific micronutrient (e.g. folate, B_12_) and macronutrient (dietary fat) with changes in temperament^(21,22)^. However, to date, no published study has looked at the associations between prenatal iodine and temperament in infancy. Moreover, many neurodevelopmental outcomes including temperament present with a sex or gender bias^(23)^. Given the focus herein on prenatal neuro-programming, we conceptualise influences of biological sex rather than the social construct of gender^(24)^. In early development, differing structure, neuronal morphology and synaptic connections lead to sexually dimorphic brain circuitry^(25)^. Sex steroids differentially influence kinetics and toxicity of chemicals and micronutrients^(26)^. Sex differences in antioxidant defence and placental responses also play a role^(27)^. Although data are sparse in relation to prenatal iodine intake, recent findings from the preschool years suggests that associations may differ early in infancy based on child sex^(28)^.

We leveraged an ethnically diverse pregnancy cohort in the Northeastern USA to examine relations among prenatal iodine intake (dietary and supplemental) and infant temperament. Given the potential for sexual dimorphism in neurodevelopment in relation to maternal nutrition^(29,30)^, sex-specific effects were also examined.

## Methods

### Study participants

Participants were from the PRogramming of Intergenerational Stress Mechanisms (PRISM) study, an ongoing longitudinal pregnancy cohort aimed at exploring relationships among prenatal social, nutritional and chemical environmental factors and child developmental outcomes. PRISM recruited 1691 women receiving prenatal care from the Beth Israel Deaconess Medical Center and East Boston Neighborhood Health Center in Boston, MA (March 2011–December 2013) and The Mount Sinai Hospital in New York City, NY (April 2013–April 2020). Eligibility criteria included age ≥ 18 years, English- or Spanish-speaking, and singleton pregnancy. Participants were excluded if they consumed ≥ 7 alcoholic drinks/week prior to pregnancy recognition or any alcohol consumption during pregnancy, had a positive HIV status, or congenital abnormalities that could impact participation. The analytic sample for the current study includes 892 mother–child participants with data on prenatal dietary and supplemental iodine intake.

### Maternal iodine levels

#### Dietary and supplemental iodine

The Block98 FFQ has been validated to evaluate dietary nutrient consumption among pregnant US women, including among ethnic minorities^(31)^. Maternal dietary iodine intake and supplement use over the past 3 months were assessed during the second trimester through the modified Block98 (version 98.2; NutritionQuest, Berkeley, CA, USA) FFQ (Block 2006_Bodnar FFQ), which consists of approximately 120 food/beverage items^(31)^ The Block98 FFQ accounts for American national consumption data and was designed based on the dietary questionnaire used by the third National Health and Nutrition Examination Survey (NHANES III)^(31,32)^. The FFQ contains items about typical food and beverage intakes, including how often (rarely or never, daily, weekly, monthly) and how much (small, medium or large serving with portion size pictures provided) on average they were consumed in the prior 3 months^(33)^. Additional items accounted for the type and frequency of dietary supplements used throughout the prenatal period. FFQs were administered in English or Spanish by bilingual research assistants.

To compute iodine micronutrient values in food, beverages and supplements, we used the most recent US Department of Agriculture (USDA), Food and Drug Administration (FDA), the National Institutes of Health (NIH) Office of Dietary Supplements (ODS), NIH Database for the Iodine Content of Common Foods^(34–36)^ and other scientific resources^(37)^. Supplement data were linked to NHANES dietary supplement files. For specific iodine-containing supplement use analysis, we identified supplements by their inclusion of iodine based on their ingredient identification codes in the Dietary Supplement Database (DSD) file. After matching the reported dietary supplement product with the ingredient information from the NHANES database, we categorised study participants as user or non-user of dietary supplements with iodine and matched the compositional quantity of iodine in the respective supplement. Estimates of iodine intake from dietary sources were generated through linkage to dietary intake tools and to the data generated by the NHANES. To estimate updated iodine intake data from reported foods, the database developed by USDA version 3.0^(34)^, the FDA Center for Food Safety and Applied Nutrition (CFSAN) and the NIH-ODS were utilised. We examined the correlation between food groups and dietary iodine intake finding that intake was most positively correlated with dairy products, seafood, meat and poultry, fruit and vegetable juices (canned with salt), and nuts, seeds, sweets and salty snacks. As in prior reports^(38)^, dairy product consumption had the largest positive correlation coefficient with dietary iodine intake (*r* = 0·90) compared to the other food groups where correlation ranged from *r* = 0·26 to *r* = 0·37 (*P* < 0·05 for all). Women were classified as having met or not met the EAR for iodine for pregnant women of 160 µg/d.

### Infant temperament

Women completed the Infant Behavior Questionnaire-Revised (IBQ-R) when infants were approximately 6 months old^(15,39)^. The IBQ-R has been validated in a culturally and sociodemographically diverse sample in the USA among infants 3–12 months old^(15,39)^, with good internal consistency. Frequency of engagement in and reactions to specific day-to-day behaviours over the prior 1 to 2 weeks were reported by mothers, with response options ranging on a seven-item Likert scale from 1 = never to 7 = always. The IBQ-R has fourteen individual scales including Distress to Limitations, Fear, Duration of Orienting, Smiling and Laughter, High Intensity Pleasure, Soothability, Falling Reactivity/Rate of Recovery from Distress, Cuddliness, Perceptual Sensitivity, Sadness, Approach, Vocal Reactivity, Activity Level and Low Intensity Pleasure. The scales were used to calculate three overall infant temperament dimensions: Surgency/Extraversion, Negative Affectivity, and Orienting/Regulation, consistent with prior work^(15)^ as well as in our sample based on the results of a confirmatory and exploratory factor analysis in the PRISM cohort^(39)^. *Surgency/Extraversion* included scales of Vocal Reactivity, High Intensity Pleasure, Activity Level, Perceptual Sensitivity and Smiling and Laughter; *Negative Affectivity* included scales of Distress to Limitations, Sadness, Fear, and Falling Reactivity/Rate of Recovery from Distress; and *Orienting/Regulation* included scales of Duration of Orienting, and Low Intensity Pleasure. Research has now shown this factor structure converges in multiple cultures^(40)^. Surgency/Extraversion, Negative Affectivity and Orienting/Regulation scores were derived by computing the mean of the corresponding subscale scores with higher scores indicating greater levels of that temperament domain.

### Covariates

Maternal age at birth in years, parity, maternal education, annual household income, previous treatment for a thyroid disease (yes or no), child sex and child age at temperament (IBQ-R) assessment were considered as potential covariates. Maternal age at birth was scored continuously. Parity was scored as nulliparous, primiparous or multiparous. Maternal educational attainment was categorised as completed high school/general equivalency diploma or less or completed more than high school (associate degree or some college, college, or graduate degree). Annual household income was categorised as less than $25 000/year, $25 000–50 000/year or more than $50 000/year. Treatment for thyroid disorder was based on self-report of treatment for thyroid disease in the past and from abstraction from medical records. Child sex was recorded as male and female. Child age at temperament assessment was recorded in months.

### Statistical analysis

Data analysis included mother–child participants with no missing food iodine intake (*n* 892). Missing data for infant temperament scores (33 %) and covariates (0·5–5 %) were imputed by chained equations^(41)^. A total of forty datasets were imputed using 500 between imputation iterations. Imputation diagnostic plots are presented in online Supplementary Figures 1 and 2. Descriptive statistics of frequencies and relative frequencies and means and standard deviations were used to summarise categorical and continuous variables, respectively. Next, dietary iodine and dietary/supplemental iodine levels were summarised according to maternal and child factors to assess for any differences in intake. Similarly, mean child infant temperament scores for Surgency/Extraversion, Negative Affectivity and Orienting/Regulation were summarised according to maternal and child sociodemographic factors and prenatal dietary iodine intake levels. Child infant temperament scores were normally distributed; therefore, linear regression was used to determine if prenatal total dietary iodine intake alone and diet plus supplements intake were associated with infant temperament scores, with analyses conducted separately by child sex after testing for multiplicative interaction between prenatal iodine and child sex, setting the threshold for significant interaction to *P* < 0·2. Because thirty mothers had more than one child represented in the study, regression models accounted for clustering of children within mothers. Analyses were performed in SAS v. 9.4 (SAS Institute) and R 4·3 (R Foundation for Statistical Computing).

## Results

### Sample characteristics

The sample was ethnically diverse, with 44 % of the women identifying as Black/Hispanic Black and 35 % as non-Black Hispanic. Nearly half (46 %) reported an annual household income < $25 000/year, and 40 % reported having less than a high school education. Six percent noted prior treatment for thyroid disease. For the sample overall, the median (IQR) prenatal dietary iodine intake was 161 µg/d (IQR: 110, 279). Nearly half of the women had intakes less than the EAR for pregnant women of < 160 µg/d, whether based on dietary intake (*n* 443, 49 %) or when food and supplements were considered together (*n* 379, 43 %). Table 1 summarises median iodine intake, based on both dietary intake and dietary plus supplemental intake, according to maternal and child characteristics. There were no significant differences in median iodine intake across maternal and child characteristics (Table 1).

**Table 1:** Distribution of sociodemographic characteristics according to dietary and supplemental iodine intake, PRISM birth cohort

	*n*	Mean	sd *	Percent	Food iodine (µg/d)		*P*-value‡	Food and supplemental iodine (µg/d)		*P*-value
					Median	IQR†		Median	IQR	
Overall	892			100	161	110, 279		189	116, 320	
Maternal factors										
Age at birth, mean (sd)*		29·7	5·9							
Age at birth (years)							0·9			0·6
18–< 25	209			23·4	162	107, 296		175	113, 324	
25–< 35	507			56·8	158	108, 278		192	114, 319	
≥ 35	176			19·7	164	116, 262		198	126, 320	
Gestational age at FFQ, mean (sd), weeks		27·9	14·5							
Breast-feeding duration (months), mean (sd)		6·2·	6·6							
Race/ethnicity							0·03			0·02
Non-Hispanic White	144			16·5	138	107, 208		148	110, 268	
Black/Hispanic Black	382			43·8	165	109, 291		201	115, 338	
Non-Black Hispanic	309			35·4	177	114, 301		198	126, 333	
Other	37			4·24	141	93, 262		153	107, 281	
Missing	20									
Education							0·9			0·7
> High school	521			59·9	159	113, 271		194	118, 314	
≤ High school	349			40·1	166	102, 304		188	112, 335	
Missing	22									
Annual household income							0·1			0·2
< 25 k	383			46·4	177	108, 317		199	114, 341	
25–< 50 k	208			25·2	164	109, 267		194	126, 330	
≥ 50 k	235			28·5	146	107, 245		170	112, 301	
Missing	66									
Parity							0·3			0·4
0	204			24·4	156	105, 262		198	109, 304	
1	329			39·4	156	111, 290		193	118, 349	
> 1	303			36·2	173	113, 281		195	121, 324	
Missing	56									
Thyroid disease treatment||							0·5			0·4
No	823			94	162	110, 286		192	116, 328	
Yes	53			6	158	116, 213		170	116, 286	
Missing	16									
Child factors										
Age at IBQ-R§ (years), mean (sd)		0·54·	0·12							
Sex										
Female	291			47·8						
Male	318			52·2						

PRISM, PRogramming of Intergenerational Stress Mechanisms; IQR, interquartile range; IBQ-R – Infant Behavior Questionnaire-Revised.

* sd.

† IQR.

‡ *P*-value for Wilcoxon–Mann–Whitney rank sum test.

§ IBQ-R.

|| Treatment for a thyroid disease was based on self-report and medical record extraction.

Table 2 summarises infant temperament scores across covariates. Infant temperament scores differed by maternal age at birth, race/ethnicity and income. Specifically, children of younger mothers at birth had significantly higher Extraversion/Surgency (mean = 5·36; sd: 0·65) and Negative Affectivity (mean = 3·21; sd: 0·77) scores than children of older mothers. Children of non-Black Hispanic mothers had higher Surgency/Extraversion (mean = 5·34; sd: 0·70), Negative Affectivity (mean = 3·24; sd: 0·77) and Orienting/Regulation (mean = 5·40; sd: 0·56) scores than the respective overall mean scores and higher than the scores in children of women from the other racial/ethnic groups. Children of women in the $25 000–$50 000/year income category had the highest scores for Surgency/Extraversion (mean = 5·34; sd: 0·70), Negative Affectivity (mean = 3·24; sd: 0·77) and Orientating/Regulation (mean = 5·40; sd: 0·56) when compared to the other income groups (Table 2).

**Table 2:** Infant temperament scores according to maternal characteristics and dietary iodine intake

	Surgency/extraversion		*P*-value†	Negative affectivity		*P*-value	Orienting/regulation		*P*-value
	Mean	sd *		Mean	sd *		Mean	sd *	
Overall, mean (sd)*	5·25	0·71		3·15	0·72		5·33	0·61	
Maternal factors									
Age at birth groups			0·01			0·05			0·1
18–< 25	5·36	0·65		3·21	0·77		5·39	0·59	
25–< 35	5·25	0·73		3·17·	0·71		5·29	0·63	
≥ 35	5·15	0·69		3·04	0·68		5·35	0·56	
Race/ethnicity			< 0·0001			< 0·0001			0·002
Non-Hispanic White	5·00	0·64		2·89	0·58		5·23	0·62	
Black/Hispanic Black	5·30	0·73		3·18	0·72		5·33	0·63	
Non-Black Hispanic	5·34	0·70		3·24	0·77		5·40	0·56	
Other	5·06	0·60		3·19	0·62		5·07	0·60	
Education			0·06			0·0001			0·5
≤ High school	5·31	0·72		3·27	0·78		5·34	0·61	
> High school	5·22	0·70		3·08	0·67		5·32	0·61	
Parity			0·9			0·5			0·4
0	5·25	0·69		3·15	0·67		5·35	0·58	
1	5·26	0·75		3·13	0·75		5·29	0·64	
> 1	5·25	0·68		3·19	0·72		5·35	0·57	
Thyroid disease treatment‡			0·2			0·05			0·8
No	5·26	0·70		3·17	0·72		5·33	0·61	
Yes	5·13	0·84		2·96	0·72		5·35	0·63	
Estimated average requirement (EAR) for pregnant women§			0·1			0·3			0·9
EAR < 160 µg/d	5·22	0·71		3·13	0·72		5·33	0·62	
EAR ≥ 160 µg/d	5·29	0·71		3·18	0·72		5·32	0·60	
Supplemental iodine use				0·06			0·2		0·8
No	5·22	0·72		3·17	0·72		5·32	0·62	
Yes	5·35	0·69		3·09	0·74		5·38	0·56	

* sd.

† *P*-value for ANOVA.

‡ Treatment for a thyroid disease was based on self-report and medical record extraction.

§ EAR for prenatal dietary and supplemental iodine intake.

Table 3 shows infant temperament dimension scores by prenatal iodine intake (dietary and supplements) and prior treatment for thyroid disease for boys and girls. There were no meaningful differences across groups. Similarly, results considering dietary intake of iodine alone were substantively like those using dietary plus supplemental iodine intake (data not shown).

**Table 3:** Iodine intake parameters by infant temperament scores for boys and girls

	Surgency/extraversion					Negative affectivity					Orienting/regulation				
	Girls		Boys			Girls		Boys			Girls		Boys		
Dietary iodine	Mean	sd *	Mean	sd *	*P*-value	Mean	sd *	Mean	sd *	*P*-value‡	Mean	sd *	Mean	sd *	*P*-value
Estimated average requirement (EAR) for pregnant women)†					0·2					0·4					0·9
EAR < 160 µg/d	5·22	0·72	5·21	0·69		3·16	0·73	3·10	0·71		5·32	0·62	5·34·	0·62	
EAR ≥ 160 µg/d	5·24	0·76	5·33	0·68		3·19	0·76	3·16	0·69		5·34	0·59	5·31	0·60	
Supplemental iodine use					0·1					0·2					0·9
No	5·21	0·74	5·26	0·69		3·20	0·75	3·14	0·68		5·33	0·62	5·32	0·62	
Yes	5·35	0·67	5·33	0·66		3·07	0·72	3·11	0·76		5·35	0·55	5·32	0·57	
Thyroid disease treatment§					0·3					0·1					0·9
No	5·24	0·72	5·28	0·68		3·18	0·75	3·15	0·70		5·33	0·61	5·32	0·61	
Yes	5·18	0·87	5·05	0·79		3·11	0·72	2·73	0·65		5·32	0·65	5·39	0·61	

* sd.

† EAR for prenatal dietary and supplemental iodine intake.

‡ *P*-value for ANOVA.

§ Treatment for a thyroid disease was based on self-report and medical record extraction.

Table 4 summarises the results of linear regression models examining associations between prenatal iodine intake and infant temperament dimension scores among girls and boys separately. Effect estimates of dietary iodine and dietary and supplemental iodine intake (EAR ≥ 160 µg/d compared to the referent group of EAR < 160 µg/d) were considered in separate models. The direction of effect estimates was generally similar in girls and boys, whereby higher iodine intake in pregnancy was associated with higher Surgency/Extraversion, lower Negative Affectivity and higher Orienting/Regulation scores. After adjusting for covariates, only the association between dietary plus supplemental iodine intake EAR ≥ 160 µg/d and lower Negative Affectivity scores among boys was meaningfully different (*β* = –1·66 (–1·97, –1·35)). Both prenatal dietary and dietary/supplemental iodine intake at an EAR ≥ 160 µg/d was associated with a higher Surgency/Extraversion score for girls in unadjusted models *β* = 0·23 (95 % CI = 0·13, 0·32); when adjusted for covariates, the association became less precise, *β* = 0·05 (95 % CI = –0·08, 0·10). The prenatal iodine intake by child sex interaction term was significant at *P* < 0·2 level only for Negative Affectivity when dietary/supplemental iodine intake was considered.

**Table 4:** Associations between dietary iodine with and without supplemental iodine intake and infant temperament: overall sample and by child sex strata

	Surgency/extraversion				Negative affectivity				Orienting/regulation			
	Crude		Adjusted		Crude		Adjusted		Crude		Adjusted	
	*β*	95 % CI	*β*	95 % CI	*β*	95 % CI	*β*	95 % CI	*β*	95 % CI	*β*	95 % CI
Overall sample												
Food iodine*	0·07	–0·02, 0·17	0·08	–0·02, 0·17	0·05	–0·05, 0·14	0·05	–0·05, 0·14	−0·01	–0·09, 0·07	−0·01	–0·09, 0·07
Food and supplemental iodine	0·08	–0·02, 0·17	0·09	–0·01, 0·18	0·02	–0·07, 0·12	0·03	–0·07, 0·12	−0·002	–0·08, 0·08	−0·002	–0·08, 0·08
Girls												
Food iodine	**0·23**	**0·13, 0·32**	0·03	–0·10, 0·16	−0·08	–0·26, 0·10	0·03	–0·10, 0·16	0·04	–0·14, 0·23	0·01	–0·10, 0·12
Food and supplemental iodine	**0·23**	**0·13, 0·33**	0·05	–0·08, 0·19	−0·08	–0·26, 0·10	−0·04	–0·17, 0·09	0·04	–0·14, 0·23	0·02	–0·09, 0·13
Boys												
Food iodine	0·12	–0·01, 0·25	0·12	–0·02, 0·25	0·002	–0·15, 0·16	0·01	–0·15, 0·16	−0·02	–0·13, 0·09	0·04	–0·08, 0·15
Food and supplemental iodine	0·10	–0·03, 0·23	0·10	–0·03, 0·23	0·06	–0·08, 0·20	**−1·66**	**–1·97, –1·35**	−0·01	–0·12, 0·10	0·09	–0·01, 0·19

EAR, estimated average requirement; IOM, Institute of Medicine.

Bold font delineates significant results.

* Food Iodine and Food and Supplemental Iodine levels based on the IOM, EAR for pregnant women ≥ 160 µg/d compared to referent group EAR < 160 µg/d. Models were adjusted for maternal age at birth, education, parity and previous treatment for a thyroid disease.

## Discussion

This study examined associations between iodine intake during pregnancy and infant temperament in an urban sample of women and children in the Northeastern USA. We found that nearly half of these women reported iodine intake during pregnancy below the EAR of ≥ 160 µg/d whether based on dietary intake (49 %) or when food and supplements were considered together (43 %), similar to recent estimates reported in another US cohort of pregnant women finding that 41 % remained below the EAR even after supplementation^(42)^. Prenatal iodine intake from diet plus supplements at or exceeding an EAR ≥ 160 µg/d was associated with lower negative affectivity scores after adjusting for covariates, particularly among boys.

Infant temperament may have long-term consequences for development, including influencing later personality and social skills development and risk for emotional and behavioural problems^(15)^. It is notable that we found a benefit of adequate iodine intake in pregnant women on reduced negative affectivity in 6-month-old boys. It has been suggested that features of negative affectivity (fear, sadness, distress reactivity and recovery) may have import for developmental outcomes. Measures of infant negative affectivity show similarity to measures of negative affectivity in older children and to the adult personality factor of neuroticism^(15)^, suggesting that negative affectivity may be relatively stable across the life course. A meta-analysis^(20)^ found negative affectivity before age 24 months to be positively associated with subsequent psychopathology, including ADHD and autism spectrum disorder, and internalising and externalising problems, assessed prior to 18 years old. Further, they found that higher self-regulation was associated with lower risk of later psychopathology, including internalising problems and ADHD, but not autism spectrum disorder. Thus, explicating factors that shape negative affectivity may inform our understanding of the earliest origins of mental health risk, resilience and other developmental outcomes.

Although we found associations between more optimal iodine intake when considering dietary and supplemental use together and reduced infant negative affectivity specifically in boys, research has been mixed on the benefits of iodine supplementation during pregnancy. Some studies have indicated evidence of no protective effect of supplemental iodine use during pregnancy on neurodevelopment and psychopathological outcomes examined in older children^(3)^. The Norwegian Mother and Child Cohort Study reported no beneficial effects of maternal use of iodine-containing supplementation on child ADHD diagnosis or symptom score^(8)^. Further, initiation of iodine supplementation among iodine-deficient mothers in the first trimester of pregnancy was associated with an increased risk of ADHD in this Norwegian sample^(8)^. Murcia et al. (2011) found that maternal intake of iodine-containing supplements was associated with lower psychomotor achievement in infants, supporting the controversial nature of iodine supplementation^(5)^. A systematic review of randomised controlled trials in populations with severe iodine deficiency found that iodine supplementation prior to or early in pregnancy reduced the risk of congenital hypothyroidism and improved motor function, but no effects on cognition were reported in preschool-aged children^(43)^. Likewise, a randomised controlled trial in mild-to-moderately deficient populations also reported no differences in preschool child behaviour or cognition (verbal and performance IQ) scores between the iodine supplementation group and placebo^(44)^. Therefore, our findings of significantly lower adjusted negative affectivity score among boys warrant further exploration.

Such discrepancies may in part be explained by findings that associations between maternal iodine intake in pregnancy and neurodevelopmental outcomes in children are complex and may be nonlinear^(45)^. Furthermore, our finding may have been due to over-adjustment or non-adjustment for unmeasured confounders. Additional research is needed to elucidate these inconsistencies before clinical recommendations can be made based on child neurodevelopmental outcomes.

### Strengths and limitations

Our study has several strengths and makes a unique contribution to this literature. The examined associations have been understudied in general and specifically among ethnically mixed US samples. Indeed, few studies have examined this association because prior to 2020, there was no US national database of the iodine content of common food items because of the high variability in iodine content of food items. We assessed both dietary and supplemental intakes to characterise prenatal iodine intake and to the best of our knowledge, this is the first study to examine prenatal iodine intake in relation to infant temperament. The focus on behavioural domains could identify children early in development who may be at risk for longer-term behavioural and psychological disorders and thus is a strength, as research on the neurodevelopmental effects of prenatal iodine intake have largely focused on outcomes in later childhood.

We also consider some limitations. Food and supplemental intake estimates were based on participant recall of usual intake over the previous 3 months and therefore may be subject to potential measurement error. For example, food items and choices available in an FFQ may not be as precise as dietary data collected by food records or 24-h dietary recalls where specific brand name products can be reported. Although the high daily variation in iodine intake may limit reliance on a 24-h dietary recall, it is useful when used in combination with FFQ data. Indeed, research has shown that FFQ are a useful and less expensive option for obtaining estimates of usual intake over time (e.g. months), including during pregnancy, with less participant burden^(46)^. Also, FFQ can better capture day-to-day variation as they assess food items that may be less frequently or episodically consumed. Moreover, misclassification of nutrient intake is likely random and therefore would be expected to result in an underestimation of associations. Nevertheless, future research could be enhanced through validation with food records and/or 24-h dietary recalls or the addition of biomarkers (e.g. UIC). Notably UIC is not without its limitations, for instance, analysis of a single spot urine only reflects iodine content of recently consumed food, whereas recommendations for considering repeated UIC over gestation is likely to overcome this limitation. In addition, measures of thyroid hormones will be useful to consider in future studies for a more accurate assessment of habitual iodine intake. Considerations need to be made for major food groups of iodine (e.g. seafood, eggs and dairy products) in future studies. Although we had fifteen iodine food groups, we were unable to adjust for them individually due to our limited sample size. Lack of adjustment for iodine food groups may have resulted in measurement error. Although the recently created USDA Database of iodine in food items account for iodised salt in cooked and processed food, it is difficult to accurately estimate iodine intake from iodised salt used in cooking, food processing and at the table^(47)^. As effects of iodine on infant temperament might be modest, future research in larger samples may detect meaningful associations beyond those found in our sample as well as provide greater power to examine sex-specific effects suggested in these results. Infant temperament was ascertained through maternal report. Maternal report of infant temperament leverages the mother’s ability to observe her infant’s behaviour over a range of contexts, which is an advantage; however, maternal factors (e.g. personality and psychopathology) may influence reporting accuracy. The IBQ-R was designed to reduce the influence of such biases by inquiring about multiple examples of concrete infant behaviours for each domain rather than asking for abstract judgements^(15)^. Furthermore, maternal ratings of infant negative affectivity on the IBQ-R correlate with laboratory observations^(48)^, although others report discrepancies^(49)^. Although parental perception of temperament may reveal subtle differences according to cultural background^(50)^, we have previously documented construct validity in this ethnically diverse sample^(39)^. Future studies should also consider relative contribution of other important factors contributing to neurobehavioural outcomes (e.g. genetics) along with nutritional factors such as iodine as well as gene x nutrition interactions.

### Conclusions

A significant proportion of the ethnically diverse women in this US sample reported suboptimal iodine intake in pregnancy, consistent with concerning trends from national surveillance data^(9)^ and recent findings reported in another US cohort of pregnant women^(42)^. Suboptimal iodine intake in pregnancy may have implications for child neurodevelopment, evident as early as infancy, as shown in this study. Identifying risk and protective factors that are readily amenable to intervention such as optimising prenatal nutritional intake of essential micronutrients such as iodine can inform prevention strategies.

## Supporting information

Akinkugbe et al. supplementary material Akinkugbe et al. supplementary material

## Supplementary material

For supplementary material accompanying this paper visit https://doi.org/10.1017/S1368980024001575

## References

[1] Bath SC (2019) The effect of iodine deficiency during pregnancy on child development. Proc Nutr Soc 78, 150–160. doi: 10.1017/s0029665118002835. 30642416

[2] Zimmermann MB (2009) Iodine deficiency. Endocr Rev 30, 376–408. doi: 10.1210/er.2009-0011. 19460960

[3] Abel MH , Caspersen IH , Meltzer HM et al. (2017) Suboptimal maternal iodine intake is associated with impaired child neurodevelopment at 3 years of age in the Norwegian mother and child cohort study. J Nutr 147, 1314–1324. doi: 10.3945/jn.117.250456. 28515161

[4] Dominguez-Perles R , Gil-Izquierdo A , Ferreres F et al. (2019) Update on oxidative stress and inflammation in pregnant women, unborn children (nasciturus), and newborns – Nutritional and dietary effects. Free Radic Biol Med 142, 38–51. doi: 10.1016/j.freeradbiomed.2019.03.013. 30902759

[5] Murcia M , Rebagliato M , Iñiguez C et al. (2011) Effect of iodine supplementation during pregnancy on infant neurodevelopment at 1 year of age. Am J Epidemiol 173, 804–812. doi: 10.1093/aje/kwq424. 21385833

[6] Hatch-McChesney A & Lieberman HR (2022) Iodine and iodine deficiency: a comprehensive review of a re-emerging issue. Nutrients 14, 3474. doi: 10.3390/nu14173474. PMC9459956 36079737

[7] Kerver JM , Pearce EN , Ma T et al. (2021) Prevalence of inadequate and excessive iodine intake in a US pregnancy cohort. Am J Obstet Gynecol 224, 82 e1–82 e8. doi: 10.1016/j.ajog.2020.06.052. PMC7779669 32653458

[8] Abel MH , Ystrom E , Caspersen IH et al. (2017) Maternal iodine intake and offspring attention-deficit/hyperactivity disorder: results from a large prospective cohort study. Nutrients 9, 1239. doi: 10.3390/nu9111239. PMC5707711 29137191

[9] Caldwell KL , Pan Y , Mortensen ME et al. (2013) Iodine status in pregnant women in the National Children’s Study and in U.S. women (15–44 years), National Health and Nutrition Examination Survey 2005–2010. Thyroid 23, 927–937. doi: 10.1089/thy.2013.0012. PMC3752509 23488982

[10] Perrine CG , Herrick K , Serdula MK et al. (2010) Some subgroups of reproductive age women in the United States may be at risk for iodine deficiency. J Nutr 140, 1489–1494. doi: 10.3945/jn.109.120147. 20554903

[11] Perrine CG , Herrick KA , Gupta PM et al. (2019) Iodine status of pregnant women and women of reproductive age in the United States. Thyroid 29, 153–154. doi: 10.1089/thy.2018.0345. 30351199 PMC7984425

[12] WHO (2024) Urinary Iodine Concentrations for Determining Iodine Status Deficiency in Populations. Vitamin and Mineral Nutrition Information System. https://iris.who.int/bitstream/handle/10665/85972/WHO_NMH_NHD_EPG_13.1_eng.pdf?sequence=1 (accessed 2 January 2024).

[13] Griebel-Thompson AK , Sands S , Chollet-Hinton L et al. (2023) A scoping review of iodine and fluoride in pregnancy in relation to maternal thyroid function and offspring neurodevelopment. Adv Nutr 14, 317–338. doi: 10.1016/j.advnut.2023.01.003. 36796438 PMC10229380

[14] Rothbart MK & Ahadi SA (1994) Temperament and the development of personality. J Abnorm Psychol 103, 55–66. doi: 10.1037//0021-843x.103.1.55. 8040481

[15] Gartstein MA & Rothbart MK (2003) Studying infant temperament via the revised infant behavior questionnaire. Infant Behav Dev 26, 64–86.

[16] Kraybill JH , Kim-Spoon J & Bell MA (2019) Infant attention and age 3 executive function. Yale J Biol Med 92, 3–11. 30923468 PMC6430162

[17] Enlow MB , Devick KL , Brunst KJ et al. (2017) Maternal lifetime trauma exposure, prenatal cortisol, and infant negative affectivity. Infancy 22, 492–513. doi: 10.1111/infa.12176. 28983193 PMC5624542

[18] Klein DN , Dyson MW , Kujawa AJ et al. (2012) Temperament and internalizing disorders. In Handbook of Temperament, pp. 541–561 [ M Zentner and RL Shiner , editors]. New York, NY: The Guilford Press.

[19] Abulizi X , Pryor L , Michel G et al. (2017) Temperament in infancy and behavioral and emotional problems at age 5·5: the EDEN mother-child cohort. PLoS One 12, e0171971. doi: 10.1371/journal.pone.0171971. 28199415 PMC5310866

[20] Kostyrka-Allchorne K , Wass SV & Sonuga-Barke EJS (2020) Research review: do parent ratings of infant negative emotionality and self-regulation predict psychopathology in childhood and adolescence? A systematic review and meta-analysis of prospective longitudinal studies. J Child Psychol Psychiatry 61, 401–416. doi: 10.1111/jcpp.13144. 31696514

[21] Lipton LR , Brunst KJ , Kannan S et al. (2017) Associations among prenatal stress, maternal antioxidant intakes in pregnancy, and child temperament at age 30 months. J Dev Orig Health Dis 8, 638–648. doi: 10.1017/s2040174417000411. 28651674 PMC6075665

[22] Gustafsson HC , Kuzava SE , Werner EA et al. (2016) Maternal dietary fat intake during pregnancy is associated with infant temperament. Dev Psychobiol 58, 528–535. doi: 10.1002/dev.21391. 26709151 PMC5026407

[23] Khan B & Avan BI (2020) Behavioral problems in preadolescence: does gender matter? Psych J 9, 583–596. doi: 10.1002/pchj.347. 32061151

[24] Mauvais-Jarvis F , Bairey Merz N , Barnes PJ et al. (2020) Sex and gender: modifiers of health, disease, and medicine. Lancet 396, 565–582. doi: 10.1016/S0140-6736(20)31561-0. 32828189 PMC7440877

[25] Cosgrove KP , Mazure CM & Staley JK (2007) Evolving knowledge of sex differences in brain structure, function, and chemistry. Biol Psychiatry 62, 847–855. doi: 10.1016/j.biopsych.2007.03.001. 17544382 PMC2711771

[26] Morris JA , Jordan CL & Breedlove SM (2004) Sexual differentiation of the vertebrate nervous system. Nat Neurosci 7, 1034–1039. doi: 10.1038/nn1325. 15452574

[27] Minghetti L , Greco A , Zanardo V et al. (2013) Early-life sex-dependent vulnerability to oxidative stress: the natural twining model. J Matern Fetal Neonatal Med 26, 259–262. doi: 10.3109/14767058.2012.733751. 23020682

[28] Goodman CV , Hall M , Green R et al. (2022) Iodine status modifies the association between fluoride exposure in pregnancy and preschool boys’ intelligence. Nutrients 14, 2920. doi: 10.3390/nu14142920. PMC9319869 35889877

[29] Bale TL , Baram TZ , Brown AS et al. (2010) Early life programming and neurodevelopmental disorders. Biol Psychiatry 68, 314–319. doi: 10.1016/j.biopsych.2010.05.028. 20674602 PMC3168778

[30] Ceasrine AM , Devlin BA , Bolton JL et al. (2022) Maternal diet disrupts the placenta-brain axis in a sex-specific manner. Nat Metab 4, 1732–1745. doi: 10.1038/s42255-022-00693-8. 36443520 PMC10507630

[31] Brunst KJ , Kannan S , Ni YM et al. (2016) Validation of a food frequency questionnaire for estimating micronutrient intakes in an urban US sample of multi-ethnic pregnant women. Matern Child Health J 20, 250–260. doi: 10.1007/s10995-015-1824-9. 26511128 PMC4959268

[32] Wirfält AK , Jeffery RW & Elmer PJ (1998) Comparison of food frequency questionnaires: the reduced Block and Willett questionnaires differ in ranking on nutrient intakes. Am J Epidemiol 148, 1148–1156. doi: 10.1093/oxfordjournals.aje.a009599. 9867258

[33] Cade J , Thompson R , Burley V et al. (2002) Development, validation and utilisation of food-frequency questionnaires – a review. Public Health Nutr 5, 567–587. doi: 10.1079/phn2001318. 12186666

[34] Roseland JM , Spungen JH , Patterson KY et al. (2022) USDA, FDA, and ODS-NIH Database for the Iodine Content of Common Foods. Release 3; 2023. https://www.ars.usda.gov/ARSUSERFILES/80400535/DATA/IODINE/IODINE%20DATABASE_RELEASE_3_REVISION_1_DOCUMENTATION.PDF (accessed December 2023).

[35] Ershow AG , Haggans CJ , Roseland JM et al. (2022) Databases of iodine content of foods and dietary supplements-availability of new and updated resources. J Acad Nutr Diet 122, 1229–1231. doi: 10.1016/j.jand.2022.03.017. 35378333

[36] Pehrsson PR , Roseland JM , Patterson KY et al. (2022) Iodine in foods and dietary supplements: a collaborative database developed by NIH, FDA and USDA. J Food Compost Anal 109, 104369. doi: 10.1016/j.jfca.2021.104369. PMC9365050 35967902

[37] Ershow AG , Skeaff SA , Merkel JM et al. (2018) Development of databases on iodine in foods and dietary supplements. Nutrients 10, 100. doi: 10.3390/nu10010100. PMC5793328 29342090

[38] Lee KW , Shin D , Cho MS et al. (2016) Food group intakes as determinants of iodine status among US adult population. Nutrients 8, 325. doi: 10.3390/nu8060325. PMC4924166 27240399

[39] Bosquet Enlow M , White MT , Hails K et al. (2016) The Infant Behavior Questionnaire-Revised: factor structure in a culturally and sociodemographically diverse sample in the United States. Infant Behav Dev 43, 24–35. doi: 10.1016/j.infbeh.2016.04.001. 27088863 PMC4909571

[40] Shiner RL , Buss KA , McClowry SG et al. (2012) What is temperament now? Assessing progress in temperament research on the Twenty-Fifth Anniversary of Goldsmith et al.(). Child Dev Perspect 6, 436–444.

[41] White IR , Royston P & Wood AM (2011) Multiple imputation using chained equations: issues and guidance for practice. Stat Med 30, 377–399. doi: 10.1002/sim.4067. 21225900

[42] Griebel-Thompson AK , Sands S , Chollet-Hinton L et al. (2023) Iodine intake from diet and supplements and urinary iodine concentration in a cohort of pregnant women in the United States. Am J Clin Nutr 118, 283–289. doi: 10.1016/j.ajcnut.2023.04.005. 37407165 PMC10493429

[43] Zhou SJ , Anderson AJ , Gibson RA et al. (2013) Effect of iodine supplementation in pregnancy on child development and other clinical outcomes: a systematic review of randomized controlled trials. Am J Clin Nutr 98, 1241–1254. doi: 10.3945/ajcn.113.065854. 24025628

[44] Gowachirapant S , Jaiswal N , Melse-Boonstra A et al. (2017) Effect of iodine supplementation in pregnant women on child neurodevelopment: a randomised, double-blind, placebo-controlled trial. Lancet Diabetes Endocrinol 5, 853–863. doi: 10.1016/s2213-8587(17)30332-7. 29030199

[45] Sullivan TR , Best KP , Gould J et al. (2024) Too Much too little: clarifying the relationship between maternal iodine intake and neurodevelopmental outcomes. J Nutr 154, 185–190. doi: 10.1016/j.tjnut.2023.09.008. 37716605

[46] Blumfield ML , Hure AJ , Macdonald-Wicks L et al. (2013) A systematic review and meta-analysis of micronutrient intakes during pregnancy in developed countries. Nutr Rev 71, 118–132. doi: 10.1111/nure.12003. 23356639

[47] Skeaff SA (2012) Assessing iodine intakes in pregnancy and strategies for improvement. J Trace Elem Med Biol 26, 141–144. doi: 10.1016/j.jtemb.2012.04.015. 22626584

[48] Gartstein MA & Marmion J (2008) Fear and positive affectivity in infancy: convergence/discrepancy between parent-report and laboratory-based indicators. Infant Behav Dev 31, 227–238. doi: 10.1016/j.infbeh.2007.10.012. 18082892 PMC2329820

[49] Stifter CA , Willoughby MT & Towe-Goodman N (2008) Agree or agree to disagree? Assessing the convergence between parents and observers on infant temperament. Infant Child Dev 17, 407–426. doi: 10.1002/icd.584. 19936035 PMC2779030

[50] Gartstein MA , Gonzalez C , Carranza JA et al. (2006) Studying cross-cultural differences in the development of infant temperament: People’s Republic of China, the United States of America, and Spain. Child Psychiatry Hum Dev 37, 145–161. doi: 10.1007/s10578-006-0025-6. 16874564

