# Supplementary material for: Prenatal iodine intake and infant temperament in a multiethnic US cohort
Source: Public Health Nutr. 2024 Nov 6;27(1):e226. doi: 10.1017/S1368980024001575 (PMC11645109; doi:10.1017/S1368980024001575)

**TITLE:** Prenatal iodine intake and infant temperament in a multiethnic US cohort

**SUPPLEMENTAL MATERIAL**


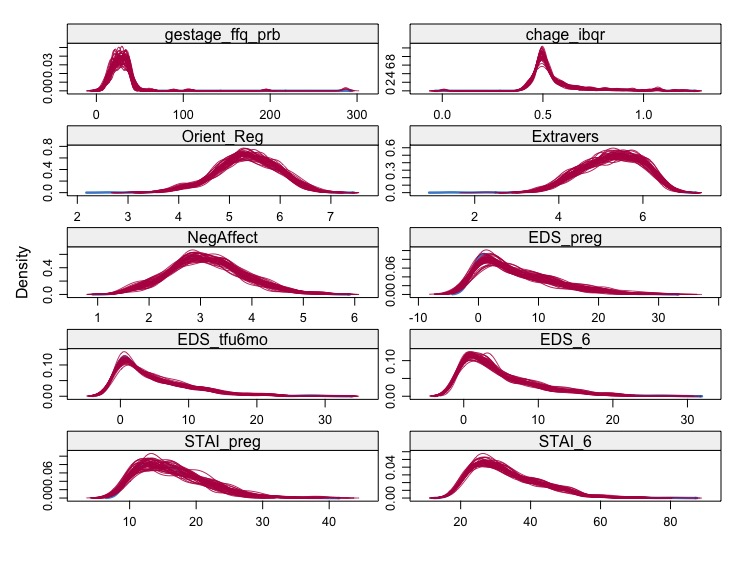
**Supplementary Figure 1.** Density plots showing distribution of observed data (blue) and imputed data (red).

**Supplementary Figure 2**. Trace plots for mean values and standard deviations of imputed variables for 500 between imputation iterations.


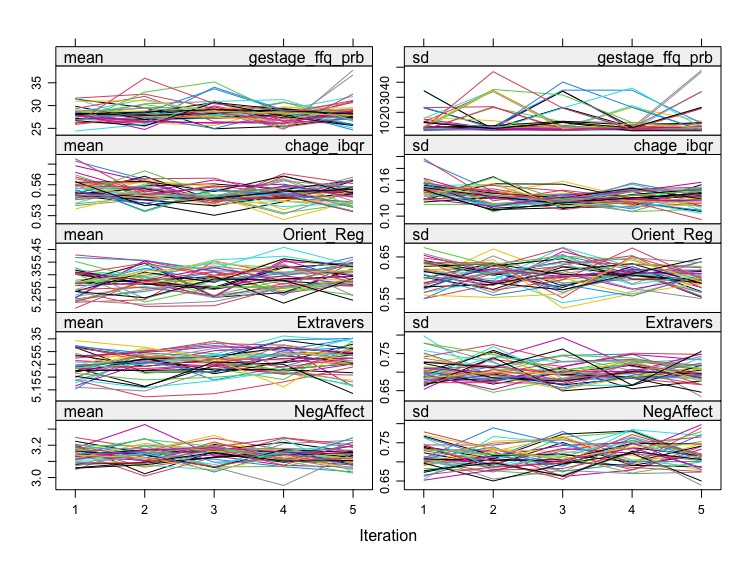

Supplement: Akinkugbe et al. supplementary material [file S1368980024001575sup001.docx]
